# Gold nanoparticles stabilized with sulphonated imidazolium salts in water and reverse micelles

**DOI:** 10.1098/rsos.170481

**Published:** 2017-07-19

**Authors:** Gustavo A. Monti, Gabriela A. Fernández, N. Mariano Correa, R. Darío Falcone, Fernando Moyano, Gustavo F. Silbestri

**Affiliations:** 1 Departamento de Química, Universidad Nacional de Río Cuarto, Agencia Postal no. 3, C.P. X5804BYA Río Cuarto, Argentina; 2 INQUISUR, Departamento de Química, Universidad Nacional del Sur (UNS)-CONICET, Av. Alem 1253, B8000CPB, Bahía Blanca, Argentina

**Keywords:** gold nanoparticles, sulphonated imidazolium salts, reverse micelles, AOT

## Abstract

Herein we describe the synthesis of gold nanoparticles (Au-NPs) in presence of sulphonated imidazolium salts [1,3-bis(2,6-diisopropyl-4-sodiumsulfonatophenyl)imidazolium (**L1**), 1-mesityl-3-(3-sulfonatopropyl)imidazolium (**L2**) and 1-(3-sulfonatopropyl)imidazolium (**L3**)] in water and in a confinement environment created by reverse micelles (RMs). The Au-NPs were characterized—with an excellent agreement between different techniques—by UV-vis spectroscopy, transmission electron microscopy (TEM), dynamic light scattering (DLS) and zeta potential. In homogeneous media, the Au-NPs interact with the imidazolium ring and the sulphonate groups were directed away from the NPs' surface. This fact is responsible for the Au-NPs' stability—over three months—in water. Based on the obtained zeta potential values we assume the degree of coverage of the Au-NPs by the imidazolium salts. In *n*-heptane/sodium 1,4-bis (2-ethylhexyl) sulfosuccinate (AOT)/water RMs, the Au-NPs formed in presence of sulphonated imidazolium salts present different patterns depending on the ligand used as stabilizer. Interestingly, the Au-NPs are more stable in time when the salts are present in AOT RMs (three weeks) in comparison with the same RMs system but in absence of ligands (less than an hour). Clearly, the sulphonated imidazolium salts are very effective Au-NPs stabilizers in a different medium and this generates a plus to be able to use them for multiple purposes.

## 1. Introduction

Nanoparticles (NPs) have become popular as sustainable alternatives to conventional materials due to their chemical and physical properties, and also to their potential applications in several scientific fields [1–4]. At nanoscale, a large fraction of atoms is concentrated on the NPs' surface, playing an important role in their physical and chemical properties. The surface atoms exhibit an incomplete valence, leaving external sites available to interact with donor/acceptor species, or ligands. Therefore, NPs may exhibit similar behaviour to their corresponding metal complexes [5].

In recent years, the interest in the synthesis of gold nanoparticles (Au-NPs) for various applications in organic reactions has been increased [6–11]. The main applications of these systems are oxidation reactions [12–14], hydrogenation reactions [15] and cross-coupling reactions [16–18]. Also, El-Sayed *et al.* [19] undertook a review to provide insights on the design, synthesis, functionality and applications of Au-NPs in biomedicine and discuss their tailored interactions with biological systems to achieve improved patient health. On the other hand, Au-NPs have an additional interest; under the influence of electromagnetic radiation, electrons from the surface atoms can easily move through vacant orbital. The coherent oscillations of those electrons in resonance with the light frequency, give rise to localized surface plasmon resonance (SPR) [20], which can be used in a wide range of applications in chemistry, biology and nanotechnology [21–23].

A brief review shows us the growing interest in NP generation and stabilization. In 1951, Turkevich *et al.* [24] reported citrate reduction in aqueous medium; two decades later Frens [25] improved this methodology. Brust *et al.* [26], in 1995, proposed a two-phase method, dissolving the gold salt in water and transferring to an organic phase with thiols *via* a transfer agent suitable phase such as tetraoctylammonium bromide. Finally, Araki *et al.* [27] have made changes to this methodology; they have used *tert*-dodecanethiol, to separate the organic phase and precipitate the NPs with methanol. These particles can be then dispersed in toluene and used for further functionalization. Other synthetic methodologies such as electrochemical, sonochemical, thermal, photochemical or microwave assisted have been employed [28–39].

The use of *N*-heterocyclic carbene (NHC) as ligands has grown substantially to stabilize metallic NPs. For example, MacLeod & Johnson [40] reported the synthesis of NHC ligands conjugated with poly (ethylene glycol) for Au-NPs stabilization. These ligands were found to be compatible in biologically relevant conditions, resulting in potential biomedical applications of NHC anchored on the NPs' surfaces. De Jesús *et al.* [41] reported the synthesis and stabilization of Pt-NPs by direct decomposition of complex NHC-Pt(0) containing the sulphonated group. Glorius *et al.* [42] reported the synthesis of—highly stable in water—Pd- and Au-NPs functionalized with sulphonate- and carboxylate-NHC to study the olefine hydrogenation reaction; and recently, de Jesús *et al.* reported the first observation of Knight shift—by ^13^C NMR—for an NHC ligand, demonstrating their coordination to the surface of metal NPs [43].

It is important to mention that Nome *et al.* [44] prepared Pd-NPs in water by using sulphonated imidazolium salts as surfactants to catalyse the hydrogenation of cyclohexene, with successful results, able to be employed up to four times without loss of catalytic activity. Tatumi & Fujihara [45] have reported the first example of Au-NPs—stable in aqueous solutions—modified with a zwitterionic liquid consisting of an imidazolium cation and a sulphonate anion, with interesting perspective in catalytic and bioanalytical applications. These NPs were stabilized by non-chemical interaction between the imidazolium salts and the NPs' surface, probably electrostatic one (note: for imidazolium compounds, it is known that stabilization of gold nanoparticles is associated with an interaction with the imidazolium cationic portion, not the counteranion) [46].

It is noteworthy that reverse micelles (RMs) are considered as nanotemplate and have been used to generate NPs, allowing a good control of shape and size, resulting in narrow size dispersion [47]. RMs are supramolecular assemblies formed by surfactant molecules dissolved in non-polar solvents [48]. The encapsulated water inside RMs shows different physico-chemical properties in comparison with homogeneous media [48,49]. Perhaps one of the most used anionic surfactants for the formation of different NPs is sodium bis (2-ethylhexyl) sulfosuccinate (AOT) [50–54] We have demonstrated that in RMs the confined water has unique properties in comparison with homogeneous media and these had implication in different reactions [55,56] as well as in the generations of NPs [49,57].^,^ Many factors affect the size and form of NPs and one important factor is the concentration of the reducer. In this regard, hydrazine (N_2_H_4_) showed a strong effect on the nanoparticle morphology [58–62].

**Scheme 1.**
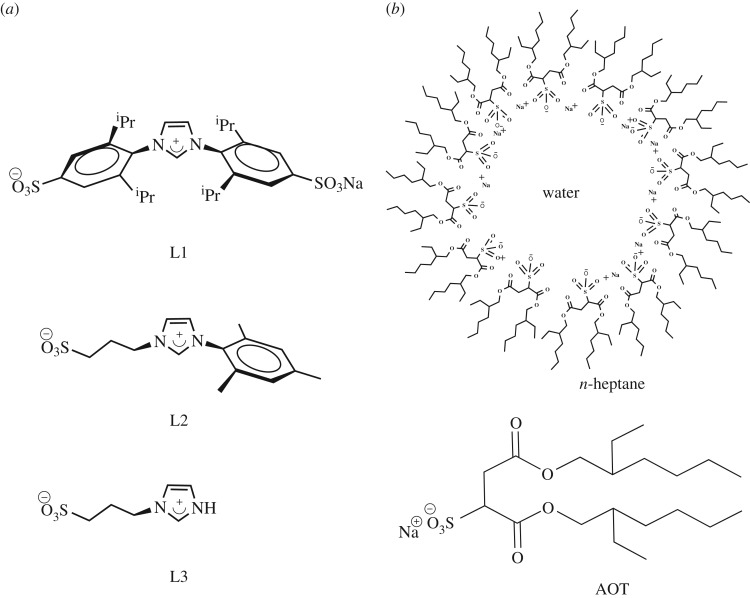
(*a*) Sulphonated imidazolium salts; (*b*) *n*-heptane/AOT/water RMs and surfactant AOT.

In addition, the possibility of obtaining extremely stable Au-NPs, even in aqueous medium and RMs, opened the doors for new applications of this metal in life sciences [63]. The main interest reported in this area is related to its use as antimicrobial agents, antifungal, antibacterial and as drug delivery vehicles [20,64–67]. Herein, we describe the synthesis—easy, rapid and reproducible to carry out—and characterization of Au-NPs stabilized by sulphonated imidazolium salts [1,3-bis(2,6-diisopropyl-4-sodiumsulfonatophenyl)imidazolium (**L1**), 1-mesityl-3-(3-sulfonatopropyl)imidazolium (**L2**) and 1-(3-sulfonatopropyl)imidazolium (**L3**), scheme 1*a*] in water and in *n*-heptane/AOT/water RMs (scheme 1*b*).

## 2. Material and methods

*n*-Heptane (Hp) from Merck, HPLC grade, was used without further purification. Sodium 1,4-bis (2-ethylhexyl) sulfosuccinate (AOT) (Sigma greater than 99% purity) was used as received and to minimize water absorption it was kept under vacuum over P_2_O_5_. Ultrapure water was obtained from Labonco equipment model 90901-01. Tetrachloroauric acid (HAuCl_4_, Sigma-Aldrich) as precursor and hydrazine monohydrate (N_2_H_4_·H_2_O, Sigma-Aldrich) as reducing agent, both for the synthesis of Au-NPs were used as received.

### 2.1. Synthesis of imidazolium salts

**Scheme 2.**
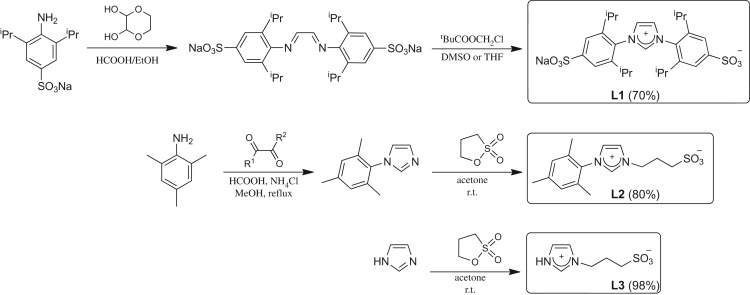
Synthesis of sulphonated imidazolium salts.

All imidazolium salts (**L1**–**L3**) were prepared and characterized according to reported procedures: **L1** [68], **L2** [69,70] and **L3** [71] as shown in scheme 2 (spectroscopy data, see electronic supplementary material).

### 2.2. Synthesis of Au-NPs in different media

Homogeneous media: A stock solution of metal precursor ([HAuCl_4_] = 0.06 M) was prepared in acetonitrile (Sintorgan, HPLC quality). Following, an appropriate amount of this solution—to obtain a given concentration (1 × 10^−4^ M) of the metal precursor—was transferred to a volumetric flask, and the acetonitrile was evaporated by bubbling dry N_2_; then, 1 × 10^−4^ M aqueous solution of sulphonated-imidazolium salt (**L1–L3**) was added. Hydrazine was dissolved in the same aqueous solution. Hydrazine concentration was 1 × 10^−3^ M when mixed (without stirring) with the metal precursor solution. The time course of the reaction was 3 s. Then, when the Au-NPs were formed, the suspensions turned pink along the time.

Reverse micelles: The Au-NPs were synthesized following the methodology described by López-Quintela [72]. The *n*-heptane/AOT/water RMs were prepared by mass and volumetric dilution. To obtain optically clear solutions, they were placed in an ultrasonic bath and the water amount was added using a calibrated microsyringe. The amount of water present in the system is expressed as the molar ratio between the polar solvent and the surfactant, *W* = [H_2_O]/[surfactant]. One solution of AOT RMs containing HAuCl_4_ dissolved at [AOT] = 0.1 M and *W* = 6, and another containing hydrazine dissolved in RMs with the same AOT concentration and *W* = 6 were prepared. The reduction process takes place by mixing the two AOT RMs systems by magnetic agitation at room temperature. The concentrations of metal ions and reducing agent based on the volume of aqueous solution, were kept constant at molar ratio [HAuCl_4_] : [N_2_H_4_] = 1 : 10.

### 2.3. General

UV/visible spectra were recorded using a spectrophotometer Shimadzu 2401 with a thermostatted sample holder at 25°C.

Transmission electron microscopy (TEM) micrographs were recorded by using a PHILIPS CM-12 microscope at 20–120 kV with a Megaview-II Docu camera and SIS NT Docu software. For TEM analysis, a drop of Au-NPs was suspended onto copper-coated grid. The grids were dried in a desiccator for 24 h and then they were examined. Histogram Au-NPs by number (*n*) were made with ‘*ImageJ*’ program, which capture the scale of TEM micrographs. Four TEM image were selected to make each histogram.

The hydrodynamic diameter and zeta potential of Au-NPs were measured using dynamic light scattering (DLS, Delsa Nano C, Beckman Coulter) operating at 658 nm. To measure DLS and zeta potential in water, cleanliness of the cuvettes used for measurements was of fundamental importance to obtain reliable and reproducible data. Cuvettes were washed with ethanol, and then with doubly distilled water and dried with acetone. Prior to data acquisition, the samples were equilibrated in the DLS instrument for 5 min at 25°C. Multiple samples of each size were made, and 30 independent size measurements were made for each individual sample at the scattering angle of 90°. The instrument was calibrated before and during the course of experiments using several different size standards. Thus, we are confident that the magnitudes obtained by DLS measurements can be taken as statistically meaningful for all the systems investigated. The algorithm used was CONTIN and the DLS experiments showed that the polydispersity of the Au-NPs sizes were less than 5%.

## 3. Results and discussion

**Scheme 3.**
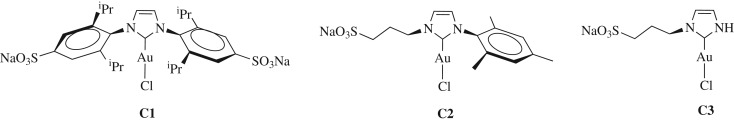
NHC-Au(I) complexes.

As was mentioned in our report, by heating aqueous solutions of **C1**–**C3**—NHC-Au(I) complexes containing the same ligands—(scheme 3) for different periods of time—depending on the structure—the solution turned pink to purplish corresponding to the generation of Au-NPs, which was confirmed by TEM microscopy.

Given our results and bibliographic reports (note: **C1** was stable in D_2_O for more than one month at room temperature and up to 3 days at 80°C. However, partial hydrolysis was observed when the solutions were heated at 100°C. On the other hand, **C2** and **C3** were not stable even at room temperature. Thus, the water-dissolution of both was accompanied—immediately—by a violet coloration [71,73]), we proceed to the Au-NPs synthesis and the study of their stabilization in the presence of sulphonated imidazolium salts **L1**, **L2** and **L3** (scheme 1*a*), in order to evaluate whether the steric bulk of the substituents influences the stability of these dispersed in water. Also, it is interesting to explore the Au-NPs formation without using the complexes route since it is very time-consuming.

### 3.1. Au-NPs stabilized with sulphonated imidazolium salts in water

**Figure 1.**
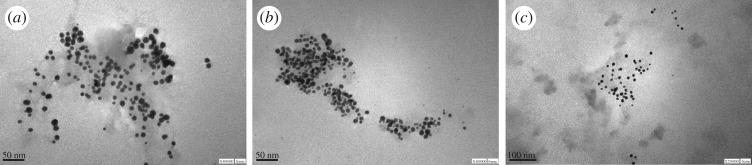
TEM image of Au-NPs stabilized with **Lx**. (*a*) **L1**; (*b*) **L2** and (*c*) **L3**.

Table 1 shows the values of SPR absorption maximum, size, and zeta potential of Au-NPs stabilized by reduction with hydrazine in the presence of **L1**, **L2** and **L3**. From table 1, the size value of the Au-NPs stabilized by **L1** obtained from DLS technique was 12 ± 2 nm. The SPR absorption spectrum of Au-NPs stabilized in presence of **L1** is located at *λ*_max_ = 545 nm (electronic supplementary material, figure S1). Figure 1*a* (TEM image) shows that the NPs have the same geometric pattern—spherical—and a size around 13 nm. Additionally, the histogram from TEM image is shown in the electronic supplementary material, figure S2. Taking into account this fact and the data shown from the TEM image (figure 1*a* and electronic supplementary material, figure S2), we can assign the large bandwidth to the larger dispersion in size.

**Table 1.** Size and zeta potential values of Au-NPs stabilized in water from DLS technique; and SPR absorption maximum ($\lambda_{\max}^{\mathrm{Abs}}$) values in water and AOT RMs from UV-visible spectroscopy.

	water				RMs
	diameter (nm)		zeta potential	$\lambda_{\max}^{\mathrm{Abs}}$	$\lambda_{\max}^{\mathrm{Abs}}$
imidazolium salts	TEM	DLS	(mV)	(nm)	(nm)
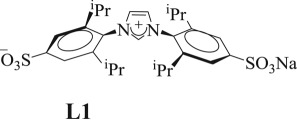	9 ± 2	12 ± 2	−21.4	545	540
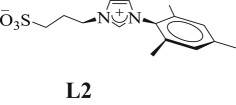	15 ± 3	20 ± 5	−37.4	518	528
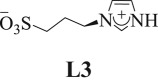	13 ± 2	13 ± 2	−21.7	521	521

The SPR of the synthesized Au-NPs in presence of **L2** is located at *λ*_max_ = 518 nm. Similar behaviour (*λ*_max_ = 521 nm) was found for **L3** (electronic supplementary material, figure S1). The hypsochromic shift (note: change of spectral band position in the absorption to a shorter wavelength, higher frequency) of the SPR provides information about their size and stabilization. According to Mie's theory [74,75], there is a relationship between SPR maximum and size. This is, the NPs are smaller when there is a hypsochromic shift in the SPR maximum. However, when the NPs are less than 20 nm, there is no correlation with Mie's theory and it is necessary to determine the size by other techniques such as TEM and dynamic light scattering.

As the figure 1*b*,*c* shows, the NPs have the same geometric shape (spherical); however, in presence of **L3** they are more disperse and their size distribution is more homogeneous than in presence of **L2** (see histograms in the electronic supplementary material, figure S2). It is important to mention that the sizes found by TEM are in agreement with the ones obtained by using DLS technique (table 1). Note, the size determined by DLS is slightly higher than TEM since here the ligand shell and the hydration layer is not present. The size of Au-NPs stabilized with **L2** is 20 ± 5 nm and 13 ± 2 nm for **L3**. The major experimental error found for **L2** confirms a more disperse and less homogeneous distribution.

**Scheme 4.**
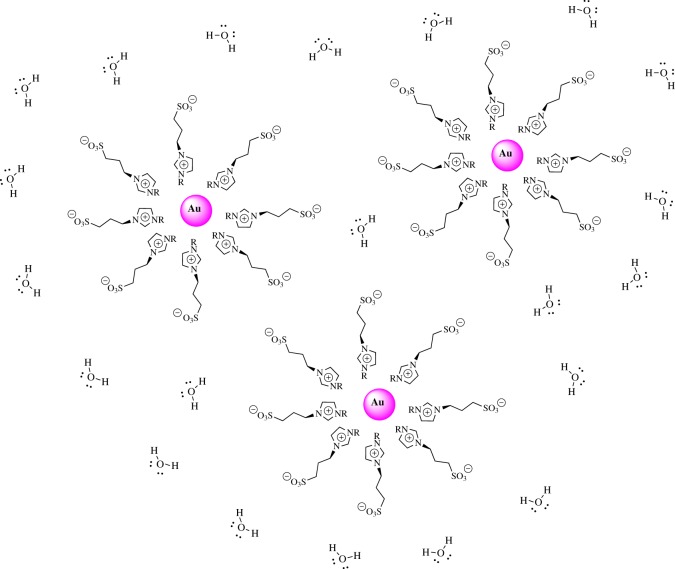
Schematic diagram of Au-NPs stabilized with imidazolium salts in water.

Taking into account the negative zeta potential values (table 1) and in agreement with the Dupont report [41], we think that Au-NPs effectively interacted with the imidazolium ring, allowing that the sulphonate groups pointed away from the NPs' surface, which are responsible for large stability in water. Scheme 4 shows a schematic representation of the Au-NPs stabilized by the imidazolium salts. Furthermore, all Au-NPs generated are stable in water for over three months at room temperature (electronic supplementary material, figure S3).

Based on zeta potential data, we estimate the NPs' coverage by ligands. Our main assumption is that Au-NPs have no interfacial charge, so the zeta potential value is due to the sulphonate charge of the different ligands. When Au-NPs were stabilized with **L2**, the zeta potential value was higher (−37.4, table 1) indicating that these are most covered. While, it is interesting to analyse the zeta potential when Au-NPs have comparable sizes (12 and 13 nm). When Au-NPs are stabilized with **L1** or **L3**, the zeta potential value is −21.4 and −21.7 respectively (table 1); however, the bulkiness of **L1** is greater than **L3** and has two sulphonate groups, therefore the value of zeta potential assigned by each sulphonate group would be half the value indicated in the table (−10.7); showing that the Au-NP stabilized with **L1** have less coverage [76].

### 3.2. Au-NPs stabilized with sulphonated imidazolium salts in AOT RMs

We reported [49] that the system formed by *n*-heptane/AOT/water at *W* = 6 can be used as stabilizing agent in the formation of Au-NPs. The stabilization was accomplished due to well-favoured droplet–droplet interactions (with *n*-heptane as non-polar solvent) and the rate of material exchange among micelles was increased, leading to a fast nucleation process in the Au-NPs formation [56]. A serious disadvantage is that Au-NPs are not stable for a long time (less than an hour). Considering that the imidazolium salts showed an excellent stabilization effect on the Au-NPs (over three months) in water, it is interesting to investigate what is the effect of the different imidazolium salts in an organized medium such as AOT RMs.

**Figure 2.**
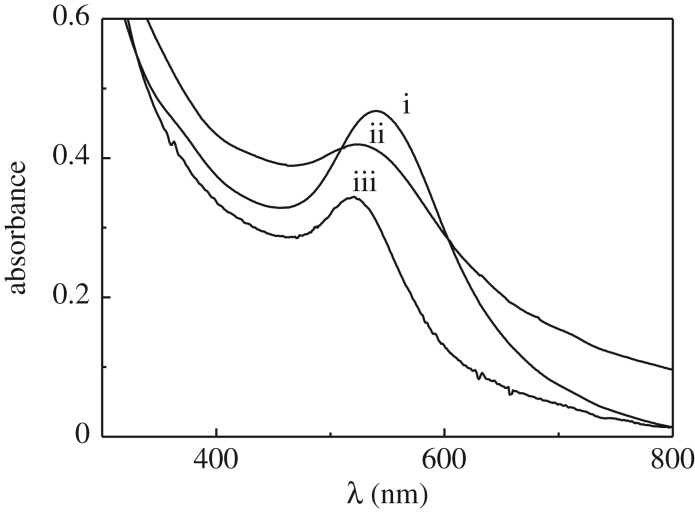
Absorption spectra of Au-NPs in presence sulphonated imidazolium salts in *n*-heptane/AOT/water RMs at *W* = 6. (i) **L1**, (ii) **L2** and (iii) **L3**. [HZ] = 3 × 10^−3^ M. [**L1**] = [**L2**] = [**L3**] = 3 × 10^−4^ M.

Figure 2 shows the absorption spectra of Au-NPs synthesized in *n*-heptane/AOT/water RMs at *W* = 6 in presence of different sulphonated imidazolium salts. As can be seen, the SPR absorption maximum generated in presence of **L1** and **L2** are located at *λ*_max_ = 540 nm and *λ*_max_ = 528 nm, respectively; while that in presence of **L3** is located at *λ*_max_ = 521 nm. The major bandwidth of the spectrum would indicate a major dispersion in size and/or aggregation of Au-NPs when these are stabilized with **L1** and **L2** in comparison with **L3**. In addition, the Gaussian band observed in presence of **L3** points out a better size distribution.

The Au-NPs were generated immediately after the addition of hydrazine independently of the employed salt, which indicates that the presence of salt does not modify the droplet–droplet interactions described above. In other words, the low concentration of salt (around 10^−4^ M) in comparison with surfactant (0.1 M), does not influence the material exchange between the metal precursor and the reducer agent. Interestingly, all Au-NPs synthesized are stable for three weeks (electronic supplementary material, figure S4) and it is due to presence of the imidazolium salt. Thus, these Au-NPs are more stable in comparison with the same RMs system but in absence of imidazolium salt (less than an hour).

**Figure 3.**
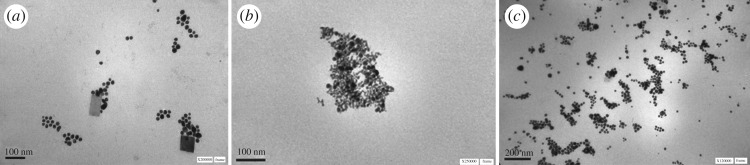
TEM images of Au-NPs in presence sulphonated imidazolium salts in *n*-heptane/AOT/water RMs at *W* = 6. (*a*) **L1**, (*b*) **L2** and (*c*) **L3**. [HZ] = 3 × 10^−3^ M. [**L1**] = [**L2**] = [**L3**] = 4 × 10^−4^ M.

Figure 3 shows the morphology and size of the Au-NPs stabilized by **L1**, **L2** and **L3**. When Au-NPs were stabilized by using **L1** (figure 3*a*) and **L2** (figure 3*b*), the particles appear like clusters of grapes and this probably takes place due to the fact that Au-NPs are more restricted in movement inside of *n*-heptane/AOT/water RMs. As it can be observed, they are spherical and are slightly agglomerated. When Au-NPs were stabilized with **L3** (figure 3*c*) in confined media, they show a good size distribution, spherical shape and practically they are not aggregated. Using the histogram for Au-NPs shown in electronic supplementary material, figure S5, the average Au-NPs size values obtained were 18 ± 4 nm, 7 ± 3 nm and 13 ± 2 nm for **L1**, **L2** and **L3**, respectively.

The obtained results suggest that the micellar system lost the strict role of ‘nano-template’ (note: the droplet size of n-heptane/AOT/water RMs at *W* = 6 is less than 5.7 nm [48]) and Au-NPs formed inside RMs are more stable in presence of the imidazolium salts. This is due to the presence of sulphonated imidazolium salts that show important interaction and good stabilization for Au-NPs. Additionally, even all the Au-NPs synthesized in AOT RMs have used the same organized media as nanoreactor (identical *W* and size), different size values of Au-NPs are obtained depending on the ligands used.

## 4. Conclusion

The aim of this work was to synthetize Au-NPs in presence of imidazolium salts in different media. The Au-NPs were characterized by DLS, TEM and zeta potential. These nanoparticles are small, spherical and monodispersed. Our results indicate that Au-NPs interact with imidazolium ring and the sulphonate groups were directed away from the NPs' surface, acting as steric barrier, avoiding aggregates and being responsible for the stability of Au-NPs dispersed in water. On the other hand—based on the zeta potential values—we can assume the degree of coverage of the Au-NPs by the imidazolium salts, showing **L3** the higher coating degree.

In *n*-heptane/AOT/water RMs, the results show two different patterns. In presence of **L1** and **L2**, Au-NPs are spherical and slightly agglomerated. Using **L3** salt as stabilizer, we obtained Au-NPs with good distribution, spherical and not aggregated. In all cases, the presence of salts increases the stabilization process over time and our studies indicate that the presence of salt does not influence the material exchange between metal precursor and reducer.

In this way, our results offer advances on organic synthesis, generating Au-NPs that are highly stable and small. This is promisingly employed in the area of catalysis and separation processes, topics that we are currently exploring in our laboratory.

## Supplementary Material

Gold Nanoparticles Stabilized with Sulfonated Imidazolium Salts in Water and Reverse Micelles

## Data accessibility

The datasets supporting this article have been uploaded as part of the electronic supplementary material.
