# Supplementary material for: Gold nanoparticles stabilized with sulphonated imidazolium salts in water and reverse micelles
Source: R Soc Open Sci. 2017 Jul 19;4(7):170481. doi: 10.1098/rsos.170481 (PMC5541566; doi:10.1098/rsos.170481)
Supplement: Gold Nanoparticles Stabilized with Sulfonated Imidazolium Salts in Water and Reverse Micelles [file rsos170481supp1.docx]

**Gold Nanoparticles Stabilized with Sulfonated Imidazolium Salts in Water and Reverse Micelles**

Gustavo A. Monti^1^, Gabriela A. Fernández^2^, N. Mariano Correa^1^; R. Darío Falcone^1^, Fernando Moyano^1,*^, Gustavo F. Silbestri^2,*^

*^1^ Departamento de Química. Universidad Nacional de Río Cuarto. Agencia Postal # 3. C.P. X5804BYA Río Cuarto. ARGENTINA.*

*^2^ INQUISUR, Departamento de Química, Universidad Nacional del Sur (UNS)-CONICET, Av. Alem 1253, B8000CPB. Bahía Blanca, ARGENTINA.*

*[fmoyano@exa.unrc.edu.ar](mailto:fmoyano@exa.unrc.edu.ar) (F. Moyano); gsilbestri@uns.edu.ar (G.F. Silbestri)

**Supplementary Material**

**Table of Contents**

| Title page and detailed list of contents of the Supporting Information. | 1 |
| --- | --- |
| ^1^H and ^13^C NMR of 1,3-bis(2,6-diisopropyl-4sodiumsulfonatophenyl)imidazolium (**L1**) | 2 |
| ^1^H and ^13^C NMR of 1-Mesityl-3-(3-sulfonatopropyl)imidazolium (**L2**) | 3 |
| ^1^H and ^13^C NMR of (3-Sulfonatepropyl)imidazolium (**L3**) | 4 |
| **Figure S1**. Absorption spectrum of Au-NPs stabilised with **Lx** in water. [**Lx**]= 1x10^-4^M. a) **L1**; b) **L2**; c) **L3**. | 5 |
| **Figure S2**. Histogram by number (*n*) for different Au-NPs stabilized with imidazolium salts. The average Au-NPs diameter for **L1** is 9 ± 2 nm, **L2** is 15 ± 3 and, for **L3** is 10 ± 2 nm from each histogram. Each histogram consists of 80 data. | 6 |
| **Figure S3.** UV-visible spectra for **L1**, **L2** and, **L3** at different months. Time (months) a: 0 , b:1, c:2, d:3. | 6 |
| **Figure S4.** UV-visible spectra at different weeks for **L1**, **L2** and **L3** in *n*-heptane/AOT /water RMs at W = 6, respectively. Time (week) a: 0 , b:1 , c:2 , d:3. | 7 |
| **Figure S5**. Histogram by number (*n*) for different Au-NPs stabilized with imidazolium salts, in *n*-heptane/AOT/water RMs at W = 6. The average Au-NPs diameter for **L1** is 18 ± 4 nm, **L2** is 7 ± 3 nm and, for **L3** is 13 ± 2 nm nm from each histogram. Each histogram consists of 70 data. | 7 |

**1,3-bis(2,6-diisopropyl-4sodiumsulfonatophenyl)imidazolium** (**L1**)

white solid; Water solubility at 25 °C: 160 g/L; ^1^H-, ^13^C-RMN (D_2_O)

**
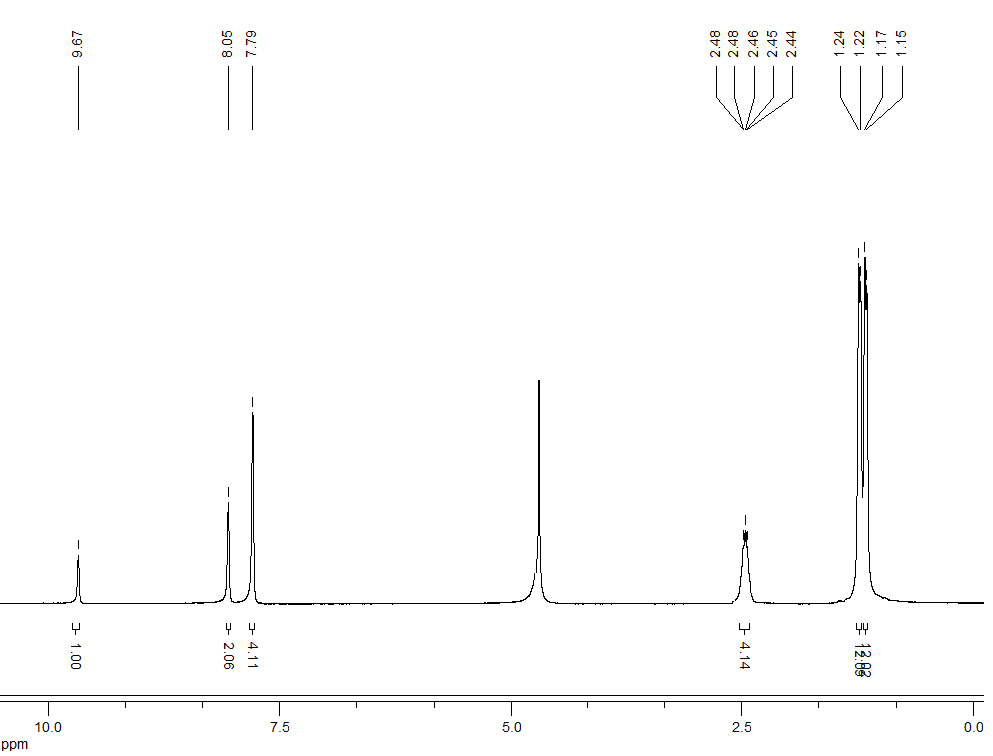
**

**
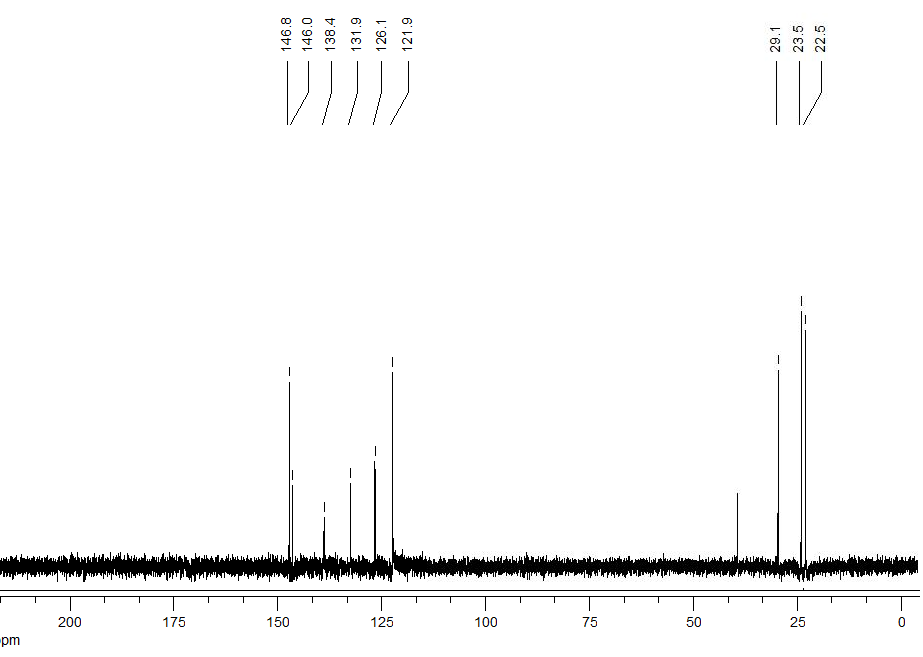
**

**1-Mesityl-3-(3-sulfonatopropyl)imidazolium** (**L2**)

white solid; Water solubility at 25 °C: 72.5 g/L; ^1^H-, ^13^C-RMN (D_2_O)

**
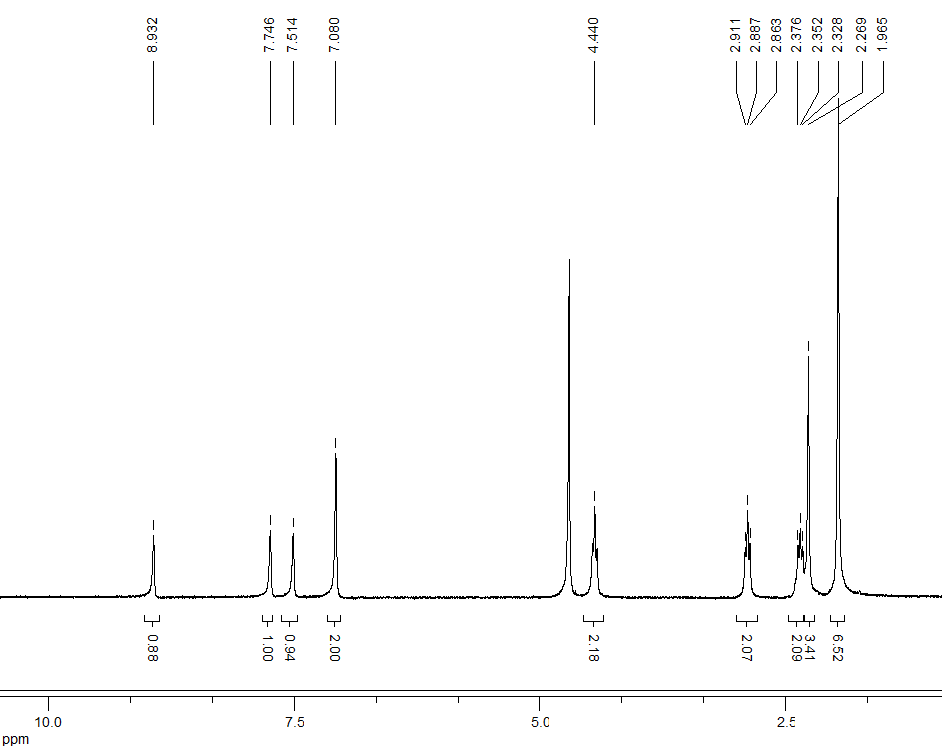
**


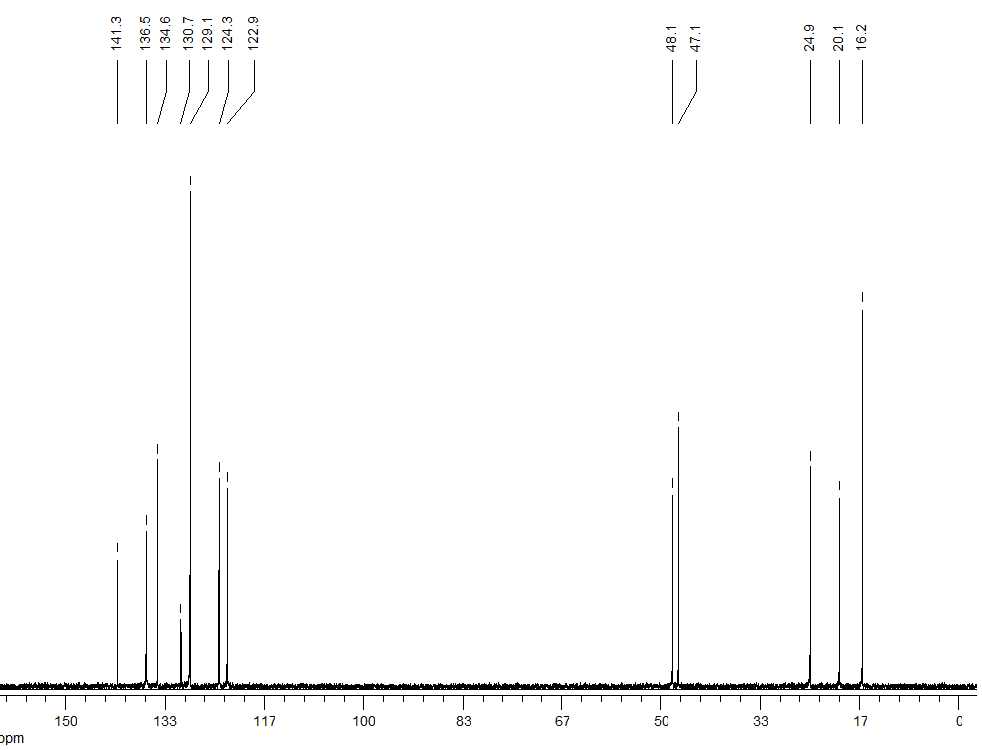


**(3-Sulfonatepropyl)imidazolium** (**L3**)

white solid; Water solubility at 25 °C: 910 g/L; ^1^H-, ^13^C-RMN (D_2_O)

**
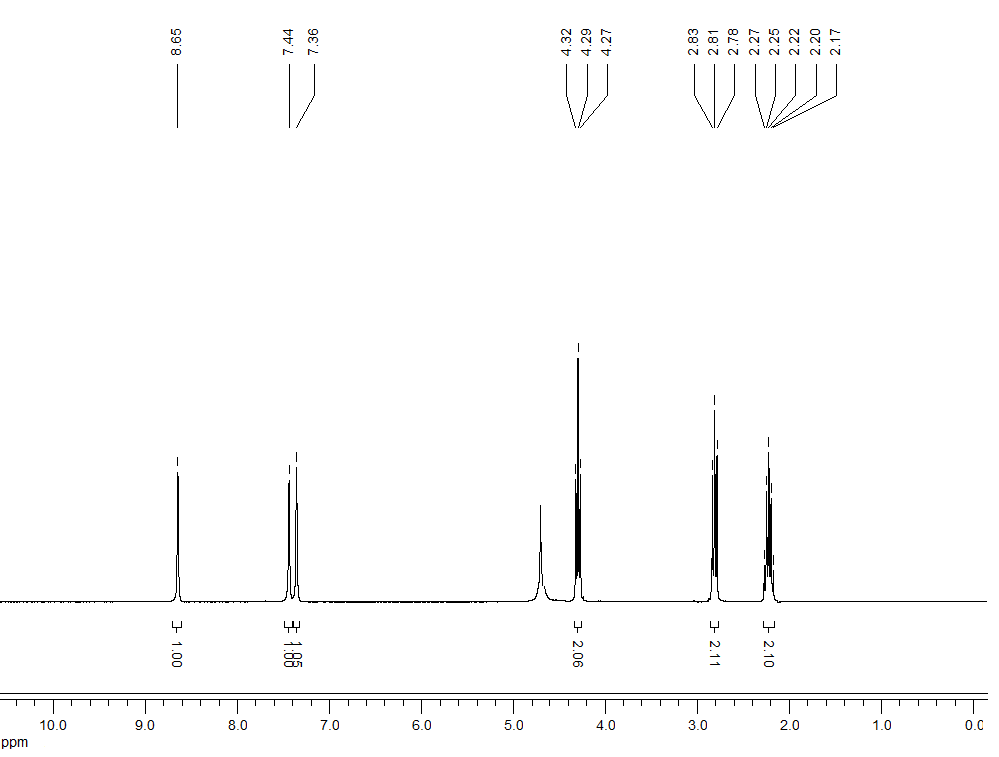
**


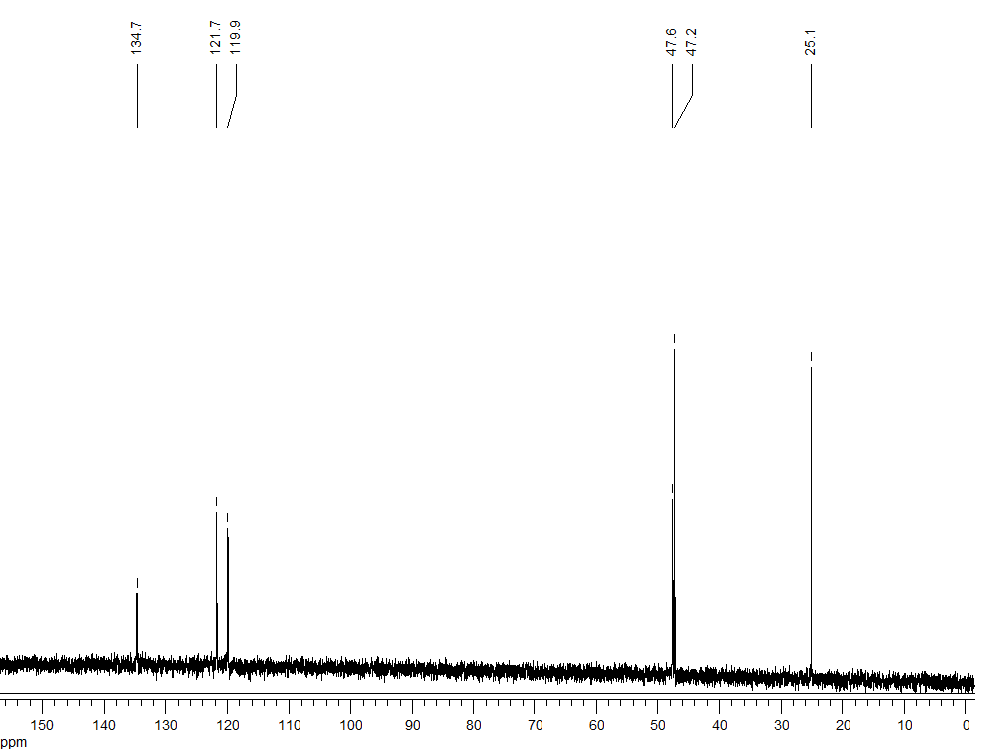

**Figure S1**. Absorption spectrum of Au-NPs stabilised with **Lx** in water. [**Lx**]= 1x10^-4^M. a) **L1**; b) **L2**; c) **L3**.

|  |  |  |
| --- | --- | --- |

**Figure S2**. Histogram by number (*n*) for different Au-NPs stabilized with imidazolium salts. The average Au-NPs diameter for **L1** is 9 ± 2 nm, **L2** is 15 ± 3 and, for **L3** is 10 ± 2 nm from each histogram. Each histogram consists of 80 data.

**Figure S3.** UV-visible spectra for **L1**, **L2** and, **L3** at different months. Time (months) a: 0 , b:1 , c:2 , d:3.

**Figure S4.** UV-visible spectra at different weeks for **L1**, **L2** and **L3** in *n*-heptane/AOT /water RMs at W = 6, respectively. Time (week) a: 0 , b:1 , c:2 , d:3.

**Figure S5**. Histogram by number (*n*) for different Au-NPs stabilized with imidazolium salts, in *n*-heptane/AOT/water RMs at W = 6. The average Au-NPs diameter for **L1** is 18 ± 4 nm, **L2** is 7 ± 3 nm and, for **L3** is 13 ± 2 nm nm from each histogram. Each histogram consists of 70 data.
